# Artificial Intelligence in Botulinum Toxin Injections: A Mini-Review of Current Applications, Challenges, and Translational Perspectives

**DOI:** 10.7759/cureus.108898

**Published:** 2026-05-15

**Authors:** Qiannan Xu, Chuanlong Jia, Xin Xia, Nan Xu, Juntian Liu

**Affiliations:** 1 Dermatology, Shanghai East Hospital, Shanghai, CHN; 2 Medical Informatics, Shanghai East Hospital, Shanghai, CHN; 3 Dermatology, Shanghai Zhimei Cosmetic Medical Clinic, Shanghai, CHN

**Keywords:** artificial intelligence (ai), botulinum injection, botulinum toxine, computer vision, dermatology dermatosurgery aesthetic medicine lasers botox clinical dermatology, multimodal learning, personalized medicine (pm), predictive modeling, three-dimensional (3d) printing, translational and precision medicine

## Abstract

Botulinum toxin (BoNT) injection is widely used in aesthetic dermatology, plastic surgery, neurology, and rehabilitation, but treatment still depends on the clinician's experience, anatomical judgment, and iterative dose adjustment. Artificial intelligence (AI) may support BoNT practice through facial analysis, anatomical mapping, treatment simulation, response prediction, image-guided injection, and objective outcome assessment. Current evidence includes chatbot-assisted planning, deep learning analysis of facial expression, magnetic resonance imaging-based prediction of dystonia response, multimodal machine learning for spasticity, computational diffusion modeling, and AI-assisted ultrasound interpretation.

This mini review, based on PubMed-indexed and related peer-reviewed literature, summarizes representative AI applications across the BoNT treatment pathway and emphasizes a cautious clinical framework in which AI supports, but does not replace, physician judgment. Evidence remains preliminary and heterogeneous, with limitations related to small sample sizes, retrospective designs, and limited external validation. However, this mini-review synthesizes the most recent and relevant high-quality studies and provides a timely, structured overview of emerging AI applications to support clinical decision-making in BoNT practice. Future translation should prioritize prospective validation, formulation-aware dose modeling, transparent governance, and physician-supervised implementation.

## Introduction and background

Botulinum toxin (BoNT) injection, expected to be a over $15 billion globally business by 2030, is a common minimally invasive procedure in aesthetic medicine and an established therapeutic option in neurology and rehabilitation [1,2]. In aesthetic practice, it is used for dynamic wrinkles, facial contouring, and adjunctive facial procedures, whereas neurological and rehabilitation indications include focal dystonia, post-stroke spasticity, and facial synkinesis [3].

Extensive high-fidelity datasets generated from film visual effects and video game simulations have already captured large-scale, detailed patterns of facial muscle movement and expression dynamics. Although botulinum toxin functions could not be recognized as a single, controllable variable that modulates muscle activity through a relatively simple and predictable dose-response relationship, it was affected by several biological factors. Complex artificial intelligence (AI) models trained on these rich movement datasets give the hope of accurately predicting and optimizing their effects, forming the core mechanistic rationale for AI integration in BoNT planning.

Despite broad adoption, BoNT injection remains operator-dependent [4]. Outcomes are influenced by anatomy, ethnicity, age, sex, muscle thickness, neuromuscular activity, product formulation, dilution, dose, volume, depth, and technique, creating challenges in planning, prediction, and complication prevention [5].

AI, including computer vision, deep learning, multimodal machine learning, large language models (LLMs), and simulation algorithms, is being explored as an adjunct to BoNT care [6,7]. These tools may quantify facial features, support treatment planning, assist image interpretation, simulate dose-response relationships, and standardize outcomes, but they should remain physician-supervised because current studies are limited by validation gaps, bias, privacy concerns, and uncertain regulatory pathways [8,9].

## Review

Search strategy and scope of review

This narrative mini-review was conducted following Preferred Reporting Items for Systematic Reviews and Meta-Analyses (PRISMA)-informed principles to enhance transparency and reproducibility, although a full systematic review and meta-analysis were not performed due to the broad, heterogeneous, and rapidly evolving nature of AI applications in BoNT therapy. Literature searches were performed in PubMed, Scopus, Embase, and Web of Science from January 2018 to May 2026 using combinations of terms: (“artificial intelligence” OR “machine learning” OR “deep learning” OR “computer vision” OR “large language model”) AND (“botulinum toxin” OR “BoNT” OR “Botox” OR “botulinum toxin type A”) AND (“injection planning” OR “aesthetic” OR “dystonia” OR “spasticity” OR “guidance” OR “prediction” OR “ultrasound”). Non-peer-reviewed examples were retained only as background concepts and were not used as primary evidence for clinical recommendations. Conference abstracts, preprints, non-English studies, case reports, and nonclinical AI studies were all excluded due to the novelty of this area.

Core AI technologies relevant to botulinum toxin injection

The most relevant AI technologies for BoNT treatment are computer vision, deep learning-based prediction, multimodal learning, image-guided segmentation, and language-model-based decision support [7,10]. Computer vision can automate landmark detection, wrinkle segmentation, asymmetry measurement, and treatment documentation, while convolutional neural networks (CNNs) and transformer-based models may support standardized pre- and post-treatment comparison [11,12].

Prediction models can integrate structural magnetic resonance imaging (MRI), gait kinematics, ultrasound features, clinical scores, and treatment metadata to estimate BoNT response or functional trajectories [13,14]. Image-guided AI may improve ultrasound interpretation by segmenting vessels, nerves, muscle layers, and target structures, although routine BoNT-specific guidance remains emerging [15,16]. LLMs may assist guideline synthesis, patient education, and preliminary plan generation, but expert review is essential because hallucinations and unsafe recommendations can occur [17].

AI in injection planning and personalized protocol development

Injection planning is an intuitive application of AI in BoNT therapy because baseline photographs can be used to identify landmarks, quantify asymmetry, estimate wrinkle severity, and generate candidate treatment plans [18]. These outputs may improve documentation and communication but cannot replace anatomical examination, informed consent, or clinician judgment [7,10].

In a comparative observational study, advanced language-vision models generated more complete and personalized facial injection plans than several comparator models, but some outputs still included inappropriate or high-risk suggestions [8]. A separate ChatGPT-supported aesthetic evaluation study suggested that AI can quantify facial harmony and post-treatment change, although clinical validation remains necessary. Table 1 summarizes concise clinical uses and safeguards for AI-assisted planning.

**Table 1:** AI-assisted planning tasks for botulinum toxin injection

Task	Potential value	Key limitation	Physician role
Facial landmark detection [8,18]	Improves baseline documentation [8,18]	Sensitive to image quality and demographic variability [19,20]	Verify and correct landmarks [8,18]
Wrinkle and symmetry quantification [11,12]	Supports objective before-and-after comparison [11,21]	May not reflect satisfaction or dynamic function [22,23]	Interpret with clinical scales and patient-reported outcomes [22,23]
Candidate injection-point generation [8]	Structures preliminary planning [8]	May suggest unsafe points without anatomical context [8]	Approve, modify, or reject all sites [8]
Dose or volume simulation [24]	Compares diffusion risk across plans [24]	Depends on tissue and formulation assumptions [5,24]	Apply product- and anatomy-specific limits [4,5]

AI in efficacy prediction and patient screening

BoNT response prediction may reduce trial-and-error treatment, lower unnecessary cost, and support referral or retreatment decisions. DystoniaBoTXNet used brain MRI and clinical variables to predict BoNT efficacy in isolated dystonia and showed high reported performance across focal dystonia phenotypes [13].

In post-stroke spasticity, the Artificial Intelligence in Managing Spasticity with Botulinum Toxin Type A (AIMS) exploratory study used an LLM-based approach to generate target-muscle and dosing recommendations, while multimodal machine learning has combined clinical and ultrasound features to predict response after ultrasound-guided BoNT and vibration therapy [25,26]. Gait rehabilitation studies have also used treatment metadata and pre-treatment kinematics to predict post-treatment trajectories [14].

A key challenge is that BoNT formulations are not interchangeable [4,5]. Product-specific units, formulation characteristics, clinical dosing conventions, diffusion behavior, and duration of action differ across formulations, so AI models trained on one product, dilution scheme, or indication may not generalize safely to another anatomical site or formulation [5,24]. Table 2 summarizes representative studies relevant to prediction, screening, and formulation-aware modeling.

**Table 2:** Selected studies relevant to AI-based response prediction and patient screening BoNT = botulinum toxin, CNN = Convolutional Neural Network, LLM = Large Language Model, AIMS = Artificial Intelligence in Muscle Spasticity

Study or authors	Context	Approach	Relevance
Yao et al. [13]	Isolated dystonia treated with BoNT [13]	Three-dimensional CNN using brain MRI and clinical variables [13]	Supports responder prediction in dystonia [13]
Filippetti et al. [26]	Post-stroke spasticity planning [26]	LLM recommendations in the AIMS exploratory study [26]	May standardize target-muscle and dose planning [26]
Chen et al. [25]	Spasticity treated with ultrasound-guided BoNT and vibration therapy [25]	Multimodal machine learning with clinical and ultrasound features [25]	Supports individualized response prediction [25]
Khan et al. [14]	Gait rehabilitation after BoNT [14]	Multi-task learning using kinematics and treatment metadata [14]	Predicts lower-limb trajectories [14]
Rahman et al. [24]	Glabellar BoNT diffusion modeling [24]	Multiscale simulation of formulation and injection volume [24]	Supports formulation-aware planning, pending clinical validation [24]

AI in injection guidance, simulation, and outcome assessment

AI may support procedural and post-procedural phases of BoNT care. In guidance, AI-enhanced ultrasound could help identify vessels, nerves, muscle borders, and injection planes, but BoNT-specific validation is still limited [27]. For cervical dystonia, ultrasound-guided BoNT treatment has clinical relevance, whereas AI-guided targeting remains exploratory [16].

Simulation models can estimate how volume, concentration, tissue properties, and formulation affect BoNT spread and may help reduce off-target diffusion, but they require prospective clinical correlation [24]. Because current AI-ultrasound integration remains largely investigational and has not yet demonstrated superiority over expert-guided ultrasound techniques. Outcome assessment is a more mature near-term use: automated photographs or videos can quantify wrinkle reduction, symmetry, movement, and emotional expression before and after treatment [21]. In facial palsy and synkinesis, three-dimensional photogrammetry and automated facial-expression analysis may improve objective assessment, although correlation with patient-centered outcomes remains incomplete [22,23]. Table 3 summarizes procedural and outcome-assessment applications [27].

**Table 3:** AI-supported guidance, simulation, and outcome-assessment applications

Application	Evidence direction	Clinical promise	Validation need
AI-enhanced ultrasound [15,27]	Segmentation of vessels, muscle layers, or target structures [15,27]	Improves anatomical awareness and training support [15,27]	Real-time accuracy across devices and operators [15,16]
Dose-response simulation [24]	Models diffusion by volume and formulation [24]	Supports individualized volume optimization [24]	Prospective linkage to outcomes and adverse events [5,24]
Photographic wrinkle analysis [11,21]	Quantifies expression or wrinkle change [11,21]	Provides objective documentation [11,21]	Validation across skin types, age groups, cameras, and lighting [19,20]
Three-dimensional photogrammetry and expression analysis [22,23]	Measures symmetry, synkinesis, and dynamic expression [22,23]	Improves reproducible outcome assessment [22,23]	Correlation with clinician scales and patient-centered outcomes [22,23]

Commercial AI software and patient-facing simulation tools

Commercial aesthetic simulation platforms increasingly claim to provide photo-based previews of BoNT or filler outcomes [18]. These tools may support communication and expectation management, but most are not independently validated as medical decision tools and should not be presented as predictors of individual outcomes [7,28]. Their use also raises concerns about unrealistic expectations, facial-image privacy, commercialization of decision-making, and bias in aesthetic datasets [10].

Challenges and limitations

Clinical translation of AI in BoNT therapy is limited by study design, data quality, explainability, privacy, and regulation [7,9]. Most studies are small, retrospective, single-center, or exploratory, and many lack external validation across institutions, populations, imaging devices, and BoNT products [8,25]. Simulation studies are useful for hypothesis generation but cannot establish safety or efficacy alone [24,28].

Dataset representativeness is especially important in aesthetic and facial-analysis applications [19,20]. Training datasets may underrepresent Asian facial anatomy, male facial aesthetics, older patients, darker skin tones, post-paralysis facial asymmetry, and neurological disability, which can produce inequitable recommendations in underrepresented groups [19,20]. Besides, the difference generated from a static photo or animation database should be noticed.

Explainability, accountability, privacy, and regulatory classification also remain unresolved [9,10]. Clinicians need to understand why a system recommends a muscle, injection point, dose range, or retreatment interval, and liability is unclear when AI contributes to an adverse event [9,10]. Because AI-assisted BoNT planning may use facial images, videos, ultrasound data, and clinical records, informed consent, secure infrastructure, and transparent governance are essential [10,20]. AI-enabled software used for medical decision-making may fall under medical-device or software-as-a-medical-device (SaMD) frameworks, and high-risk medical AI requires risk management, data quality, transparency, and human oversight [29,30].

Many predictive AI models could be vulnerable to overfitting due to small datasets and limited external validation. Future work should therefore prioritize larger and more diverse datasets, integration of dynamic facial analysis, and long-term longitudinal follow-up to improve model robustness and clinical applicability.

Future directions

The most realistic near-term direction is physician-supervised AI-assisted precision care rather than autonomous BoNT injection [7,9]. Priorities include standardized facial and ultrasound datasets, validated landmark and wrinkle analysis, formulation-aware dose simulation, objective outcome measurement, and prospective multicenter studies [12,30].

Socioeconomic bias, image acquisition bias, and device variability across smartphones and clinic equipment pose significant challenges; however, this variability also presents an opportunity for developing user-specific portable imaging devices and advanced software filters to improve standardization and model robustness.

Different from the AI-sponsored robotic hair transplant, which could be recognized as paint on a paper, AI-assisted BoNT injection is more likely a 3D sculpture. This meant it might need more database and translation to meet our standard needs. Short-term achievement could be a well-constructed human-oriented AI BoNT injection system. While longer-term concepts could include multimodal monitoring, robotic assistance, three-dimensional printed microneedle arrays, and customized delivery systems [7,31]. Microneedle-based BoNT delivery has been investigated experimentally for hyperhidrosis, and three-dimensional printed hollow microneedles have been proposed as future dermatologic delivery platforms, but these approaches remain investigational rather than established alternatives to physician-administered injection [31,32].

The responsible translation of AI into clinical practice requires not only predictive performance but also transparency and rigorous validation. In this context, Explainable AI (XAI) frameworks, such as SHAP and attention-based mechanisms, have become essential for building clinician trust, particularly in complex procedures like BoNT injection planning, where understanding model reasoning is critical for patient safety [33]. Furthermore, adherence to established AI reporting standards is fundamental for ensuring scientific credibility. Guidelines such as CONSORT-AI, SPIRIT-AI, TRIPOD+AI, and DECIDE-AI provide structured frameworks for the design, reporting, and evaluation of AI interventions in healthcare [34-36]. These standards emphasize the importance of external validation, sample size justification, and clear documentation of model limitations, elements that remain insufficiently addressed in many current studies on AI-assisted facial analysis and ultrasound-guided BoNT procedures. Incorporating these methodological principles will be crucial for advancing AI from exploratory tools to clinically reliable systems that can meaningfully augment physician expertise in the future.

## Conclusions

Artificial intelligence may improve BoNT therapy by supporting assessment, planning, prediction, procedural guidance, simulation, documentation, and outcome measurement. Its strongest near-term role is as a physician-supervised tool that standardizes facial and functional assessment, improves anatomical awareness, supports formulation-aware planning, and provides objective before-and-after evaluation.

Current evidence remains preliminary and heterogeneous. Before routine clinical integration, future studies should provide prospective multicenter validation, external testing across diverse populations, transparent reporting, privacy protection, explainable outputs, and clear regulatory classification.
